# Glyconanoparticles as a platform to multimerize peptides involved in HIV entry process

**DOI:** 10.1186/1742-4690-8-S2-P18

**Published:** 2011-10-03

**Authors:** Paolo Di Gianvincenzo, Blanca Arnaiz, Silvia Ghezzi, Elisa Vicenzi, Loϊc Martin, Soledad Penadés

**Affiliations:** 1 Lab. of GlycoNanotechnology, Biofunctional Nanomaterials Unit, CIC biomaGUNE, San Sebastián, Spain; 2 Biomedical Research Networking Centre on Bioengineering, Biomaterials and Nanomedicine (CIBER-BBN), San Sebastián, Spain; 3 Viral Pathogens and Biosafety Unit Division of Immunology, Transplantation and Infectious Diseases, San Raffaele Scientific Institute, Milano, Italy; 4 CEA/SACLAY-iBiTec-S, Gif sur Yvette, France

## Background

Binding of the gp120/gp41 complex to CD4/coreceptors is cooperative, requiring three CD4 binding events, four to six coreceptors, and multiple clusters of gp120/gp41 to effectively form a fusion pore.[1] An engineered CD4 mimetic mini protein (miniCD4) [2] and the third variable region (V3) of gp120 [3] are peptides able to block HIV entry.

Gold nanoparticles covered with carbohydrates, peptides or proteins are useful tools to study biological processes where multivalence is crucial. [4] In this work we present the study of the multimerization of miniCD4and V3 peptides onto gold glyconanoparticles (GNPs).

## Materials and methods

To prepare the GNPs we used a strategy developed in our laboratory. [5,6] MiniCD4 and V3 peptides were linked to the GNPs by covalent bond or by electrostatic interaction. In the covalent bond procedure carboxyl groups on GNPs were activated with NHS/EDC to react with amine groups of lysines present in the peptides. The so-obtained peptide-GNP complexes were characterized by ^1^HNMR, UV, TEM, gel electrophoresis and MALDI and the tri-dimensional structure of the peptides on the GNPs was studied by circular dichroism (CD).

The binding of miniCD4-GNPs to gp120 and the interaction of V3-GNPs to anti-V3 monoclonal antibody 447-52D were studied by SPR using ProteOn XPR36.

The effect of V3-GNPs on the levels of coreceptors CCR5 and CXCR4 on cell surface was tested by flow cytometry after incubation with U87.CD4.CCR5 and U87.CD4.CXCR4cells.

HIV neutralization experiments were performed using TZMBL cell line stably transfected with CD4 and both CCR5 and CXCR4. Cells were incubated in the presence or absence of GNPs with IIIB or Bal virus.

## Results

The amount of peptides on GNPs depends on their nature (e.g. number of lysine and charged amino acids) and on the GNP preparation protocols. CD experiments showed that miniCD4 peptide on GNPs does not change its conformation. However, V3 coupled to GNPs changes the conformation from essentially random coil to α-helix as indicated by the CD spectra. Conjugation of miniCD4 or V3 to the GNPs did not affect the binding ability of the peptides to the selected receptors (gp120 and anti-V3 447-52D mAb, respectively) as showed by SPR sensograms.

Flow cytometry data showed that incubation of U87.CD4.CXCR4 cells with V3 peptide or V3-GNPs decrease the level of CXCR4 on cell surface, while GNP control without peptides gave CXCR4 level comparable to the untreated control. V3-GNPs decrease CXCR4 level 5 times more than free peptide. MiniCD4-GNPs inhibit the infection as well as the free peptide in preliminary HIV neutralization experiments.

## Conclusions

MiniCD4-GNPs are able to bind gp120 but no multivalent effect was observed. Experiments with V3-GNPs gave encouraging results in the binding to both coreceptor and mAb, making these GNPs interesting tools to study their immunogenic properties and the effect on coreceptor sequestration.
